# UPLC-MS based urine untargeted metabolomic analyses to differentiate bladder cancer from renal cell carcinoma

**DOI:** 10.1186/s12885-019-6354-1

**Published:** 2019-12-05

**Authors:** Zhan Wang, Xiaoyan Liu, Xiang Liu, Haidan Sun, Zhengguang Guo, Guoyang Zheng, Yushi Zhang, Wei Sun

**Affiliations:** 1 Department of Urology, Peking Union Medical College Hospital, Chinese Academy of Medical Sciences, Peking Union Medical College, Beijing, 100730 China; 2 Core facility of instrument, Institute of Basic Medical Sciences, Chinese Academy of Medical Sciences, School of Basic Medicine, Peking Union Medical College, Beijing, 100005 China

**Keywords:** Metabolomics, UPLC-MS, Biomarkers, Bladder cancer, Renal cell carcinoma

## Abstract

**Background:**

To discover biomarker panels that could distinguish cancers (BC and RCC) from healthy controls (HCs) and bladder cancers (BC) from renal cell carcinoma (RCC), regardless of whether the patients have haematuria. In addition, we also explored the altered metabolomic pathways of BC and RCC.

**Methods:**

In total, 403 participants were enrolled in our study, which included 146 BC patients (77 without haematuria and 69 with haematuria), 115 RCC patients (94 without haematuria and 21 with haematuria) and 142 sex- and age-matched HCs. Their midstream urine samples were collected and analysed by performing UPLC-MS. The statistical methods and pathway analyses were applied to discover potential biomarker panels and altered metabolic pathways.

**Results:**

The panel of α-CEHC, β-cortolone, deoxyinosine, flunisolide, 11b,17a,21-trihydroxypreg-nenolone and glycerol tripropanoate could distinguish the patients with cancer from the HCs (the AUC was 0.950) and the external validation also displayed a good predictive ability (the AUC was 0.867). The panel of 4-ethoxymethylphenol, prostaglandin F2b, thromboxane B3, hydroxybutyrylcarnitine, 3-hydroxyphloretin and N′-formylkynurenine could differentiate BC from RCC without haematuria. The AUC was 0.829 in the discovering group and 0.76 in the external validation. The metabolite panel comprising 1-hydroxy-2-oxopropyl tetrahydropterin, 1-acetoxy-2-hydroxy-16-heptadecyn-4-one, 1,2-dehydrosalsolinol and L-tyrosine could significantly discriminate BC from RCC with haematuria (AUC was 0.913). Pathway analyses revealed altered lipid and purine metabolisms between cancer patients and HCs, together with disordered amino acid and purine metabolisms between BC and RCC with haematuria.

**Conclusions:**

UPLC-MS urine metabolomic analyses could not only differentiate cancers from HCs but also discriminate BC from RCC. In addition, pathway analyses demonstrated a deeper metabolic mechanism of BC and RCC.

## Background

Genitourinary cancers include cancers of the bladder, kidney, prostate and testicles. Other genitourinary cancers, such as adrenal, penile, ureteral and urethral cancers, are relatively rare. Among these cancers, bladder cancer (BC) and renal cell carcinoma (RCC) are, respectively, the first two commonly occurring genitourinary cancers in China and the second and third most common genitourinary cancers in Europe and North America, respectively [1]. Currently, cystoscopy and cytology are the standard procedures for the initial diagnosis and recurrence of BC, but limitations exist. Cystoscopy may fail to visualize certain areas within the bladder and may also fail to detect all cancers, particularly some cases of in situ carcinoma [2]. Cytology has high specificity and selectivity for high grade tumours but fails to provide strong predictive value for low grade tumors [3]. Regarding RCC, computed tomography, magnetic resonance imaging, and positron emission tomography are commonly used imaging diagnostic techniques [4]. However, even with the combined use of the above three techniques, early tumours remain difficult to be detected because of their small size [5]. Therefore, developing novel and convenient techniques for the detection of BC and RCC with high sensitivity and specificity are urgently required.

Recently, an increasing number of studies have used metabonomic analyses to diagnose a number of pathologies [6–8] and elucidate the clinical pathogenesis of various diseases [9, 10]. Metabonomics has several major advantages, which include the readily availability and relatively ease of analysis of biofluids, such as urine and plasma, and the derived metabolite profiles are sensitive to both environmental and genomic influences affecting the pathogenesis and progression of disease [11].

Urine is a particularly suited biofluid concerning bladder cancer and renal carcinoma due to its intimate contact with the urinary system [12]. Therefore, urine metabolomics is a promising approach for BC and RCC detection and marker discovery.

There are several studies on urine metabolomics analysis for discovering bladder cancer biomarkers. In 2011, Huang et al. [13] found that a combined urinary biomarker composed of carnitine C9:1 and an unknown metabolite had high sensitivity and specificity in discriminating 27 BC patients from 32 healthy controls (HCs). In 2014, Jin et al. [14] applied LC/MS to profile urinary metabolites of 138 patients with BC and 121 control subjects. The study identified 12 putative markers that were involved in glycolysis and beta-oxidation. Wittmann et al. [15] applied LC/MS to profile urinary metabolites of 66 BC and 266 non-BC subjects. They suggested that metabolites related to lipid metabolism may be potential BC markers. In 2017, Zhou et al. [16] applied a urinary pseudo-targeted method based on GC-MS for a BC metabolomics study. The study identified a combinatorial biomarker panel consisting of four differential metabolites that could be used for diagnosing BC and early-stage BC.

Metabolomics has also been widely applied to research on renal carcinoma biomarker discovery. In 2011, Kim et al. [17] used the UHLC/MS and GC/MS platform to perform urine metabolomics against 29 kidney cancer patients and 33 control patients. The study identified 13 significantly differentially expressed metabolites. In 2016, Monteiro et al. [12] analysed the urine metabolome of 42 RCC patients and 49 controls using NMR. A 32-metabolite/resonance signature, including 2-KG, N-methyl-2-pyridone-5-carboxamide (2-Py), bile acids, galactose, hypoxanthine, isoleucine, pyruvate, succinate, etc., was able to successfully distinguish RCC patients from controls in a principal component analysis. In 2017, Falegan et al. [18] applied NMR and GC/MS platform to perform urine and serum metabolomics against 40 RCC patients and 13 benign patients. The results showed alterations in the detected levels of glycolytic and tricarboxylic acid (TCA) cycle intermediates in RCC relative to benign masses.

These studies have unveiled potential disease biomarkers in urine. However, most metabolic markers were discovered based on small pilot studies. The limited study cohort or lack of effective validation restricts further clinical applications of these biomarkers [19]. Moreover, to our knowledge, few studies have addressed the occurrence of false positives with these approaches, e.g., the diagnosis of certain types of genitourinary cancer in patients with other genitourinary cancers or urologic disorders that present similar clinical symptoms [5]. For example, patients with BC usually present with haematuria, but haematuria can also be present in patients with other genitourinary cancers. Haematuria can be a serious confounding variable. Therefore, in our study, a urine metabolomics approach using ultra-performance LC-MS (UPLC-MS) was carried out. A total of 403 urine samples, including 146 samples from patients with BC (77 patients without haematuria and 69 patients with haematuria), 115 samples from patients with RCC (94 patients without haematuria and 21 patients with haematuria) and 142 samples from sex- and age-matched healthy controls were assessed. Multivariate statistical analysis and biomarker analysis were used to discover and externally validate the biomarker panel. Previous studies have reported that haematuria may greatly affect the outcomes of metabolic analyses. Therefore, BC patients without and with haematuria were distinguished from RCC patients by biomarker panels from urine metabolomics that may be used for the differential diagnosis of BC and RCC.

## Methods

### Inclusion and exclusion criteria

The criteria for inclusion and exclusion in our research are as follows: 1) early-stage RCC patients (namely, pathological T1 and T2 stages); non-muscle invasive BC patients; and healthy controls were chosen from the health examination centre; 2) all patients were diagnosed by postoperative pathology; 3) all hepatic functions (ALT, AST, Dbil, Tbil, etc.) and renal functions (Cr, BUN, eGFR, etc.) were within the normal range; 4) none of the patients received any other kind of therapy before the operation; 5) preoperative routine examinations and the medical history collection did not suggest other malignant tumours or metabolism-related diseases, such as diabetes mellitus and hyperlipidaemia.

### Sample collection

This study was approved by the Institutional Review Board of the Institute of Basic Medical Sciences and Peking Union Medical College Hospital, Chinese Academy of Medical Sciences, and all human subjects provided informed consent before participating in this study. Both the urine samples from cancer patients and healthy controls were collected from Peking Union Medical College Hospital. Midstream urine was collected in the morning at 07:00 a.m. –09:00 a.m. after an overnight fast to eliminate the disturbance of diet. Then, all samples were immediately stored in a − 80 °C freezer and thawed on ice before analysis. A total of 403 urine samples, including bladder cancer (BC, *n* = 146), renal cell carcinoma (RCC, *n* = 115) and healthy controls (HCs, *n* = 142), were assessed.

### Sample preparation

First, each mixture made up of acetonitrile (200 μl) and urine sample (200 μl) was vortexed for 30 s and centrifuged at 14,000×g for 10 min. Samples were dried under vacuum, and the supernatant was then blended with 200 μl of 2% acetonitrile. Before being transferred to the autosamplers, 10 kDa molecular weight cut-off ultracentrifugation filters (Millipore Amicon Ultra, MA) were applied to separate urinary metabolites from larger molecules. Samples were prepared by mixing aliquots of two hundred representative samples, and the QC samples were injected every ten samples throughout the analytical run to assess the method stability and repeatability.

### UPLC-MS analysis

The Waters ACQUITY H-class LC system coupled with an LTQ-Orbitrap mass spectrometer (Thermo Fisher Scientific, MA. USA) was launched to perform the ultra-performance LC-MS analyses of urine samples. We separated urinary metabolites with a 17 min gradient on a Waters HSS C18 column (3.0× 100 mm, 1.7 μm), and the flow speed was 0.5 ml/min. Mobile phases A and B were 0.1% formic acid in H_2_O and acetonitrile, respectively. The gradient was described as follows: 0–1 min, 2% solvent B; 1–3 min, 2–15% solvent B; 3–6 min, 15–50% solvent B; 6–9 min, 50–95% solvent B; 9–9.1 min, 95–100% solvent B; 9.1–12 min, 100% solvent B; 12–12.1 min, 100–2% solvent B; and 12–17 min, 2% solvent B. The temperature of the process was 50 °C. Scans from 100 to 1000 m/z at a resolution of 60 K were used to acquire the Full MS. The automatic gain control (AGC) target was 1× 10^6^, and the maximum injection time (IT) was 100 ms. Then, UPLC targeted-MS/MS analyses of the QC sample were conducted to identify the differential metabolites. A resolution of 15 K with an AGC target of 5× 10^5^, a maximum IT of 50 ms, and an isolation window of 3 m/z was obtained. In terms of every target with higher-energy collisional dissociation (HCD) fragmentation, 20, 40 and 60 were set as the optimal collision energies.

### Data processing

Referring to the published identification strategy [20, 21], we use the Progenesis QI (Waters, Milford, MA, USA) software to analyse the data. In the Additional file 3: QI data handling and metabolite identification processes can be found. We established various statistical techniques, such as missing value estimation, log transformation and Pareto scaling; thus, the features could be more comparable in MetaAnalyst 4.0 (http://www.metaboanalyst.ca). Any variables lost in 50% samples were discarded. The significance of variables was assessed by non-parametric tests. An adjusted *P*-value (FDR) < 0.05 was regarded as significant. We use SIMCA 14.0 (Umetrics, Sweden) software to carry out pattern recognition analyses (principal component analysis, PCA; orthogonal partial least squares discriminant analysis, OPLS-DA). Any differential variables that fulfil all the limitations were considered significant: 1) fold change > 1.5; 2) adjusted *P*-value < 0.05; and 3) VIP value above 1. We used the MetaAnalyst 4.0 platform to launch a ROC analysis and an external biomarker validation to test the prediction accuracy.

### Quality control

A strict quality control assessment is of great significance for the metabolomic analysis because some other factors, such as the sample collection, preparation or even the analytic procedures, may tremendously affect the outcomes. To eliminate the technical errors involved in our study, the samples were randomly distributed in the discovery or external validation group, and our QC samples were also analysed to assess the stability. The injected QC samples in our study showed only a small variation ranging within 2 SD (Additional file 1: S1A), conforming to the stability and reproductivity of our data, as the tight clustering further demonstrated (Additional file 1: S1B). The above analysis indicated that analytical differences may arise from the internal metabolic variation within the samples rather than from the technical bias.

## Results

### Subjects

**Fig. 1:**
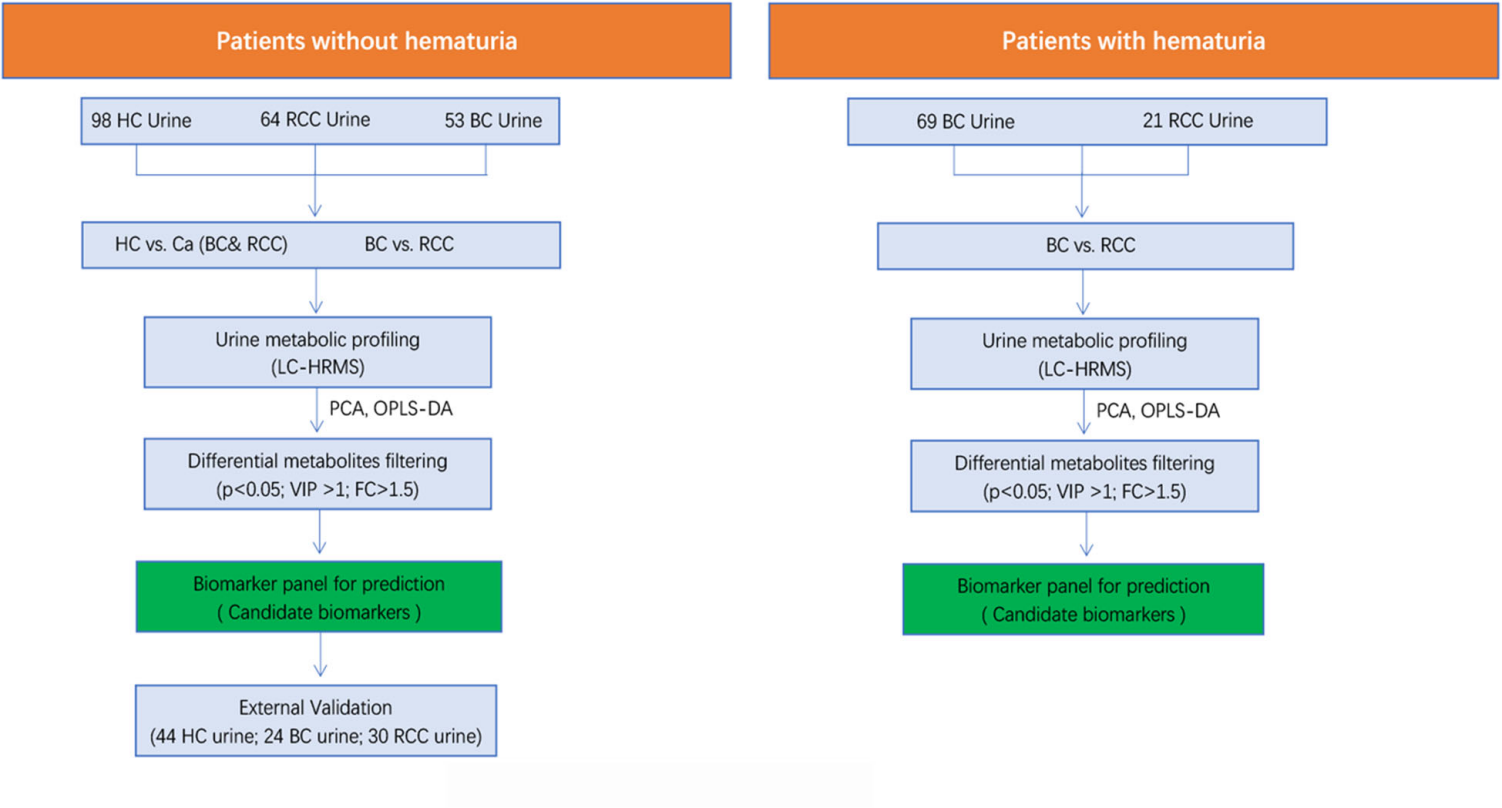
The workflow of our study

The workflow of this study is shown in Fig. 1. A total of 403 participants were enrolled in our study: 146 BC patients (77 without haematuria and 69 with haematuria), 115 RCC patients (94 without haematuria and 21 with haematuria) and 142 sex- and age-matched healthy controls. The baseline clinical information of all enrolled subjects is shown in Table 1. All the pathological diagnoses of the BC and RCC patients were confirmed after surgery by more than two professional pathologists in our hospital. Since the control samples enrolled did not have haematuria, the cancer (including BC and RCC) samples without haematuria were explored to identify cancer biomarkers. First, a pilot differential analysis on urine metabolomics was performed to discriminate cancer patients from healthy subjects. Cancer biomarkers were discovered based on metabolic profiling analysis of 98 age- and sex-matched health subjects, 53 BC patients and 64 RCC patients. The potential biomarkers were further externally validated using an independent batch of cancer patients (24 BC patients and 30 RCC patients) and 44 healthy control samples. Additionally, a pilot differential analysis was performed to discriminate urinary metabolic profiling between BC and RCC without haematuria. The potential biomarkers were further externally validated using an independent batch of 24 BC patients and 30 RCC patients. Furthermore, to find a promising biomarker panel that could distinguish BC and RCC from haematuria, the samples of 21 RCC patients and 69 BC patients with haematuria were differentially analysed (Table 2).

**Table 1:** The baseline information of all enrolled subjects in the study

Items	Without hematuria						With hematuria	
	Discovery group			Validation Group			BC	RCC
	BC	RCC	HC	BC	RCC	HC		
Cases(n)	53	64	98	24	30	44	69	21
Age(yrs)	62 (33–87)	53 (14–82)	55 (20–91)	64 (28–92)	60 (20–75)	51 (24–77)	67 (40–90)	52 (32–78)
Gender (M/F)	41/12	48/16	58/40	18/6	24/6	35/9	50/19	10/11

**Table 2:** Results of logistic regression model based on different biomarker panels

Groups	AUC	Sensitivity	Specificity
^1^Cancers(BC&RCC) vs controls			
discovery group	0.950(0.942–0.958)	0.868(0.846–0.891)	0.875(0.855–0.895)
10-fold cross-validation	0.933(0.902–0.925)	0.857(0.857–0.926)	0.880(0.822–0.939)
^2^BC vs RCC without hematuria			
discovery group	0.829(0.802–0.855)	0.832(0.801–0.862)	0.706(0.666–0.747)
10-fold cross-validation	0.784(0.695–0.874)	0.802(0.802–0.908)	0.698(0.575–0.822)
^3^BC vs RCC with hematuria			
discovery group	0.913(0.885–0.942)	0.847(0.795–0.898)	0.953(0.937–0.970)
10-fold cross-validation	0.870(0.754–0.986)	0.857(0.857–1.00)	0.913(0.847–0.980)

^1^The biomarker panel: α-CEHC, β-cortolone, deoxyinosine, flunisolide, 11b,17a,21-trihydroxypreg-nenolone and glycerol tripropanoate

^2^The biomarker panel: 4-ethoxymethylphenol, prostaglandin F2b, thromboxane B3, hydroxybutyrylcarnitine, 3-hydroxyphloretin and N′-formylkynurenine

^3^The biomarker panel: 1-hydroxy-2-oxopropyl tetrahydropterin, 1-acetoxy-2-hydroxy-16-heptadecyn-4-one, 1,2-dehydrosalsolinol and L-tyrosine

### Untargeted metabolomics could distinguish Cancer (BC & RCC) from healthy controls

**Fig. 2:**
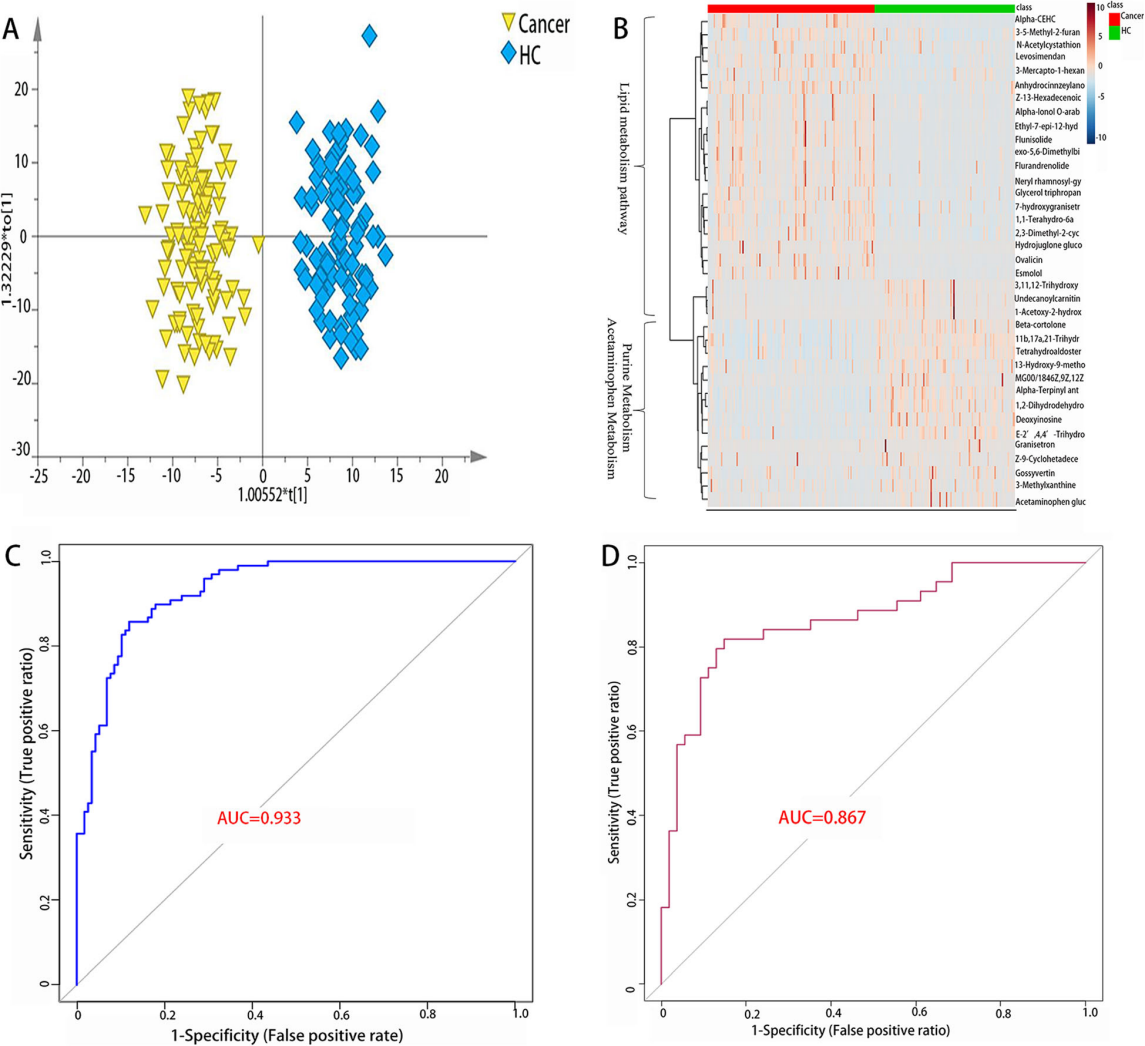
Analysis of metabolic profiling between cancers and controls. (**a**) Metabolic score plot of OPLS-DA.(**b**) Relative intensity between the cancers and controls. (**c**) ROC curve with 10-fold cross validation based on the biomarker panel. (**d**) ROC curve of external validation based on the biomarker panel

To identify the biomarkers between the cancer (BC and RCC) and healthy controls, an unsupervised PCA analysis was used to identify metabolic profiling differences. The results are shown in Additional file 2: Figure S2A. The score plot showed a significant difference between the two groups. Furthermore, to better show the difference between the cancer and healthy control groups, a supervised OPLS-DA model was launched (Fig. 2a). Based on the value of the important plot (VIP) value (VIP > 1), a total of 37 statistically differentially expressed metabolic molecules were selected (Additional file 3: Table S1a). According to Additional file 3: Table S1a, a heatmap was launched to discover the metabolic disturbance (Fig. 2b), from which we could easily draw the conclusion: compared with the healthy controls, the lipid metabolism pathway was upregulated while the purine metabolism and acetaminophen metabolism pathways were downregulated in the cancer group. To further explore the separating capacity of each metabolite, an ROC curve was applied to each molecule, and the results are presented in Additional file 3: Table S1b. As depicted in the table, 8 metabolites show a good distinctive ability, with an AUC above 0.8, along with 22 metabolites above 0.7. Furthermore, a multivariant ROC curve-based exploratory analysis (http://www.metaboanalyst.ca/faces/up-load/RocUpload View.xhtml) was used to discover the panel with the best predictive ability. As a result, a panel containing α-CEHC, β-cortolone, deoxyinosine, flunisolide, 11b,17a,21-trihydroxypreg-nenolone and glycerol tripropanoate was selected. In our testing data, the AUC was 0.95 and 0.933 for 10-fold cross-validation (Fig. 2c). Our external validation data were used to test the predictive ability of the panel, and the AUC was 0.867 (Fig. 2d).

### Untargeted metabolomics could distinguish BC from RCC without haematuria

**Fig. 3:**
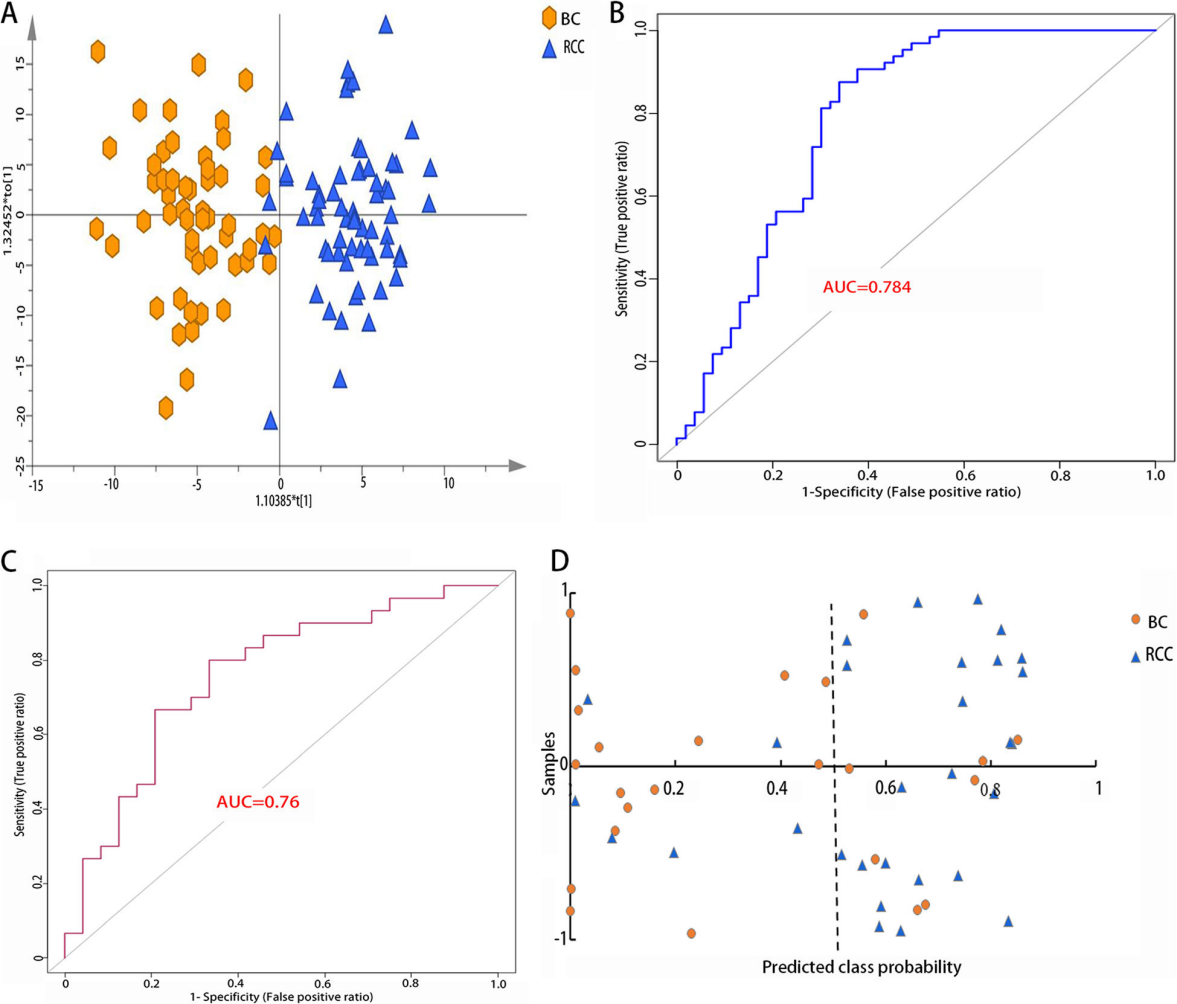
Analysis of metabolic analysis between BC and RCC without hemturia. (**a**). Metabolic score plot of OPLS-DA. (**b**). ROC curve with 10-fold cross validation based on the biomarker panel. (**c**). ROC curve of external validation based on the biomarker panel. (**d**). External prediction accuracy model based on the biomarker panel

To detect the differential metabolites between the BC and RCC groups, PCA was applied, and the results are shown in Additional file 2: Figure S2B. The picture suggested a significantly differential ability. Then, a supervised OPLS-DA model was launched (Fig. 3a), and we selected a sum of 32 metabolites with a cut-off VIP value of 1 (Additional file 3: Table S2a). The ROC curve was later used to evaluate the predictive precision. Among the differential molecules, 3 metabolites showed potential diagnostic ability with an AUC above 0.7, and 26 metabolites had an AUC above 0.6 (Additional file 3: Table S2b). The multivariant ROC curve-based exploratory analysis revealed that a metabolite panel including 4-ethoxymethylphenol, prostaglandin F2b, thromboxane B3, hydroxybutyrylcarnitine, 3-hydroxyphloretin and N′-formylkynurenine possessed the best predictive ability. The AUC under the discovery data was 0.829 and 0.784 for 10-fold cross-validation (Fig. 3b). In addition, the AUC of external validation was 0.76 (Fig. 3c). The panel showed a good ability to distinguish 16 BC patients from 24 BC patients correctly, and the rate was 24/30 for the RCC (Fig. 3d).

### Untargeted metabolomics could distinguish BC from RCC with haematuria

**Fig. 4:**
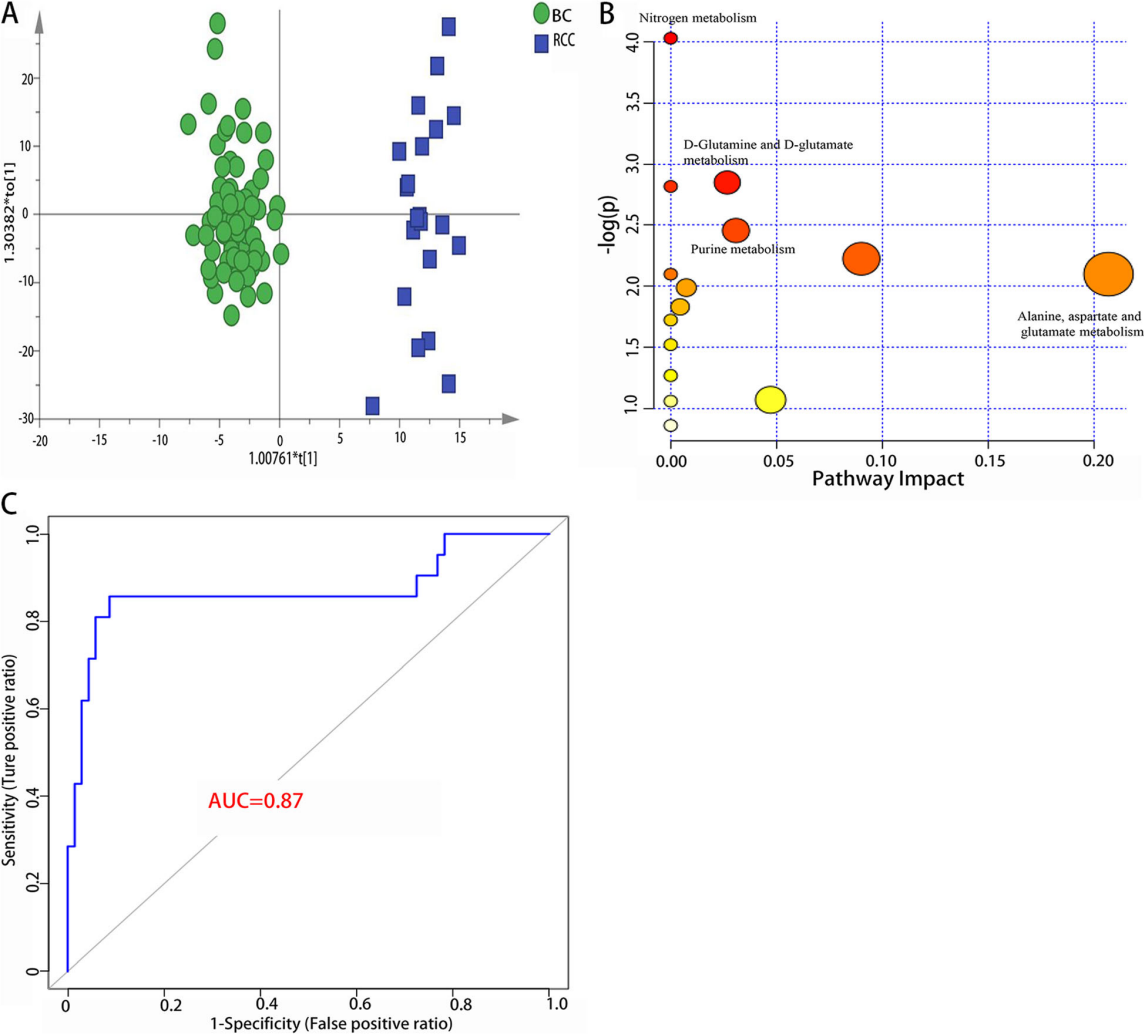
Analysis of metabolic anlysis between BC and RCC with hematuria. (**a**).Metabolic score plot of OPLS-DA. (**b**).Pathway analysis of the differential metabolites between the two subgroups. (**c**).ROC curve with 10-fold cross validation based on the metabolic biomarker panel

Similarly, a PCA analysis was first applied to explore the difference between the BC and RCC patients with haematuria, and the results are shown in Additional file 2: Figure S2C. From the figure, we could clearly observe that there was an apparent separation between the two subgroups. Then, the OPLS-DA model was structured (Fig. 4a). Based on the VIP of OPLS-DA (VIP > 1), 59 metabolic molecules in total were identified as significant differential metabolites between the two groups (Additional file 3: Table S3a). From the metabolites, it is not difficult to conclude that the metabolism concerning nitrogen metabolism, D-glutamine and D-glutamate metabolism, purine metabolism, and aspartate and glutamate metabolisms were significantly altered between the two groups. Pathway power analysis revealed that the distinguishing metabolism could aid in the separation (Fig. 4b). According to the ROC curve, 3 metabolites showed good performance in separating the BC patients from the RCC patients, with an AUC above 0.8, and the other 33 metabolites showed an AUC above 0.7 (Additional file 3: Table S3b). Further analysis indicated that a panel made up of 1-hydroxy-2-oxopropyl tetrahydropterin, 1-acetoxy-2-hydroxy-16-heptadecyn-4-one, 1,2-dehydrosalsolinol and L-tyrosine exhibited the best capacity to distinguish the independent subgroups. The AUC of the panel is 0.913 for the discovery group and 0.870 for 10-fold cross-validation (Fig. 4c).

## Discussion

Through the high-throughput measurement of endogenous metabolites, metabolomics has shown enormous prospects in discovering diagnostic cancer biomarkers in the field of renal cell carcinoma and bladder cancer. However, to the best of our knowledge, although many cancer markers have been found in bladder cancer, most studies only focused on the differentiation between cancers and healthy subjects, thus ignoring the discrimination within malignant tumours. As we know, our study is the first to explore the differential metabolites between BC and RCC patients, with or without haematuria. As a result, by comparing the BC, RCC and HCs, we found that i) a panel composed of α-CEHC, β-cortolone, deoxyinosine, flunisolide, 11b,17a,21-trihydroxypreg-nenolone and glycerol tripropanoate could well distinguish the cancer patients (BC and RCC) from the healthy controls, and this result may provide significant information about the dysregulated metabolic pathways of malignant urinary tumours. ii) a panel consisting of 4-ethoxymethylphenol, prostaglandin F2b, thromboxane B3, hydroxybutyrylcarnitine, 3-hydroxyphloretin and N′-formylkynurenine shows a good ability to differentiate BC patients from RCC patients without haematuria. iii) since previous studies have already indicated that haematuria may statistically affect the analytic outcomes of metabolomics, we also performed an exclusive experiment to certify the biomarker panel. As the result suggested, a panel comprising 1-hydroxy-2-oxopropyl tetrahydropterin, 1-acetoxy-2-hydroxy-16-heptadecyn-4-one, 1,2-dehydrosalsolinol and L-tyrosine could significantly discriminate BC patients from RCC patients among patients with haematuria.

The clustering heatmap between cancer patients and healthy controls suggested that lipid metabolism was upregulated in these cancers; this result was in accordance with the classical Warburg effect, demonstrating that cancer cells prefer to use glycolysis rather than aerobic oxidation even in the presence of oxygen [22]. The dysregulated lipid and phospholipid metabolisms showed great significance in cell mortality, cell invasion and tumour metastasis, and this result may produce enormous tumour biomarkers [23]. In previous studies, the disturbance of lipid metabolism has been reported in various studies, including BC and RCC patients. By analysing the global lipidomic profiles of 165 bladder-derived tissues, Piyarathna, et al. found that compared with benign tissues, the urothelial cancer of the bladder had higher levels of phospholipids and fatty acids and reduced levels of triglycerides, suggesting that reduced triglycerides may be used for producing energy, while the changed phospholipid may play an active role in membrane structure or signal transduction [24]. By performing comparative UPLC-MS of two isogenic human T24 bladder cancer cell lines, Young Lee et al. discovered that there was a statistically distinguished lipid species between cisplatin-sensitive and cisplatin-resistant cancer cells, suggesting that lipid-targeted new drugs may improve the prognosis of cisplatin-resistant patients [25]. For the RCC, an article reported that many fatty acids were downregulated in nonmetastatic RCC tissues as a result of overactive fatty acid oxidation. In addition, they also discovered that in metastatic RCC, lipid metabolism was upregulated, which may be related to tumour progression [26]. In some other studies, metabolites of carnitine metabolism, which are responsible for the transportation of fatty acids into the mitochondria, have been found to be increased in high-grade tumour tissues, blood serum or urine [27–29], which may be a consequence of improved fatty acid β-oxidation to sustain higher rates of cell division and growth.

In addition, 2,5,7,8-tetramethyl-2 (2′-carboxyethyl)-6-hydroxychroman (also known as α-CEHC), is an end-product of α-tocopherol, which is one group of vitamin E generated through a set of enzymatic reaction [30]. As we know, vitamin E is a potent lipid-soluble antioxidant that could help strengthen the immune system, inhibit cell proliferation and several inflammation pathways caused by infection or tumour progression [31, 32]. In our study, α-CEHC was upregulated in cancer patients compared to that in the healthy controls, and the fold change was 4.38, which confirms an accelerated vitamin E metabolism. To the best of our knowledge, our study is the first to discover the upregulation of vitamin E metabolism in bladder caners, which may be caused by inflammation secondary to tumours. Concerning renal cell carcinoma, Catchpole et al. observed an increased level of α-tocopherol in RCC tumour tissues compared with normal renal cortex tissue, consistent with the findings of Nikiforova et al. [26, 33]. In addition, analysing 66 invasive ovarian carcinomas and 9 borderline tumour tissues by gas chromatography/time-of-flight mass spectrometry, Denkert, et al. discovered that α-tocopherol 2 was elevated in cancers, and the fold change (of cancer vs borderline tumour) was 2.5 [34]. As all the vitamins in our bodies are obtained through digestion, we still cannot rule out the possibility that the increased vitamin metabolism may be just a superficial phenomenon of an increased uptake of lipids, rather than caused by cancer.

A disturbance of purine metabolism has also been detected in our study not only in the panel of cancer patients vs healthy controls but also in the group of BC vs RCC patients. However, contrary to previous studies, our research showed that compared with the controls, deoxyinosine, one of the most common precursors of DNA, was decreased in the cancer groups. In 2007, Sahu et al. enrolled 96 patients (including 72 urothelial carcinoma patients and 24 normal patients) and analysed their differential metabolites by performing UHPLC-MS/MS. As a result, both the purine and the purine metabolites were increased in urothelial cancer, suggesting the accelerated synthesis and degradation of nucleotides [35]. In a meta-analysis of 11 articles, the levels of guanine, cytosine, thymine, hypoxanthine, uracil and ribose were found to be elevated in the urine of BC patients, indicating a higher level of nucleotide metabolism [36]. Concerning the RCC, few studies have reported the differential metabolites of purine metabolism, making our study the first to demonstrate an inner mechanism. Compared with BC, the purine metabolism of RCC was upregulated slightly, which suggested a higher nucleic acid metabolism. However, it is necessary to stress that the lower purine level may be due to a much more obvious degradation together with enhanced synthesis.

N-formylkynurenine, one metabolite of the tryptophan-kynurenine pathway, was elevated in RCC compared with BC without haematuria (listed in Additional file 3: Table S2a), suggesting altered tryptophan metabolism in RCC patients. Catalysed by indoleamine 2,3-dioxygenase 1 (IDO1) and tryptophan 2,3-dioxygenases (TDO), the tryptophan (TRP) was first transformed into N-formylkynurenine (NFK) and then hydrolysed into kynurenine (KYN) by kynurenine formamidase [37]. Several studies have already revealed that IDO1 expression in a large number of cancers could lead to the depletion of TRP and accumulation of NRK and KYN, which inactivates T effector cells and thus suppresses immunity [38, 39]. The high level of NFK in the urine of RCC patients may be a symbol of local tumour immune deficiency and may facilitate tumour growth, but the deeper mechanism remains to be explored. Furthermore, a perturbation of the metabolism of other amino acids, namely, alanine, aspartate, glutamate and D- glutamine metabolism, has also been revealed between the group of cancer patients (BC vs RCC with haematuria), which suggested a distinguished protein metabolism between BC and RCC patients. In addition, elevated prostaglandin F2b and thromboxane B3 occurred in BC patients, and these molecules are biologically active signalling components of the COX and LOX pathways. The COX and LOX pathways are closely associated with the functions of inflammatory cell regulation, tumourigenesis, cell proliferation, and angiogenesis. Our results were supported by a previous metabolomic analysis of urothelial carcinoma [35], illustrating hyperactive tumour metabolism and consequent inflammation.

There also exist some limitations in our study. First, the sample scale in our study is relatively small and is single-centre study, making the data less convincing. Therefore, increasing the samples and enrolling more medical centres would be necessary in our further analyses. Second, our study focused on the discrimination of BC and RCC patients and revealed a deeper mechanism under the surface. However, due to the complete heterology of BC and RCC, it remains a question whether these cancer patients are comparable. Third, because of the epidemic differences between BC and RCC, the diagnostic age of BC patients is older than that of RCC patients, there is also a possibility that the metabolic disturbances between BC and RCC may be caused by the distinguished age between the two groups rather than by the cancer. Last but not least, due to the limitations of time and conditions, we merely used one method, metabolomics, to predict the potential altered metabolism; thus, we focused only on the small metabolites in urine. Therefore, a combination of proteomics, transcriptomics and genomics in the future could help us better understand the deeper mechanism of BC and RCC.

## Conclusions

In conclusion, based on a highly sensitive metabolomics approach, we discovered three independent early diagnostic biomarker panels that could distinguish RCC patients, BC patients and healthy controls, which may significantly benefit BC and RCC patients and thus improve their prognosis. Many altered metabolic pathways have been identified by comparative metabolomics, including lipid, vitamin E, purine, amino acid and eicosanoid metabolisms.

## Supplementary information

**Additional file 1: Figure S1.** (A).Trend plot showing the variation of t [1] over all QC Samples.(B).PC1 Versus PC2 of test samples and QC samples.

**Additional file 2: Figure S2.** Assessmentof PCA score plot between different groups. (A). Analysis of metabolic profiling between Cancers and Healthy controls. (B). Analysis of metabolic profiling between BC and RCC samples without hematuria. (C). Analysis of metabolic profiling between BC and RCC samples with hematuria.

**Additional file 3: Table S1a.** Differential metabolites between cancer(BC and RC) and healthy controls. **Table S1b.** Differential metabolites for cancer(BC and RC) distinction. **Table S2a.** Differential metabolites between BC and RC without hematuria. **Table S2b.** Differential metabolites for BC and RC without hematuria distinction. **Table S3a**. Differential metabolites between BC and RC with hematuria. **Table S3b.** Differential metabolites for BC and RC with hematuria distinction

**Additional file 4.** Data processing using Progenesis QI

## Data Availability

All the necessary materials can be found in the text or supplementary materials. Due to the privacy policy, the confidential data materials could only be obtained with the permission of the corresponding authors.

## Abbreviations

AUC: Area under the curve
BC: Bladder cancer
COX: Cyclooxygenase
GC-MS: Gas chromatography mass spectrometry
HCs: Healthy controls
IDO1: Indoleamine 2,3-dioxygenase 1
KYN: Kynurenine
LC-MS: Liquid chromatography mass spectrometry
LOX: Lipoxygenase
NFK: N-Formylkynurenine
NMR: Nuclear magnetic resonance
OPLS-DA: Othogonal partial least squares discriminant analysis
PCA: Principal Component analysis
QC: Quality control
RCC: Renal cell carcinoma
SD: Standard deviation
TDO: Tryptophan 2,3-dioxygenases
TRP: Tryptophan
UPLC-MS: Ultra-performance liquid chromatography mass spectrometry
VIP: Variable importance plots
α-CEHC: 2,5,7,8-tetramethyl-2(2′-carboxyethyl)-6-hydroxychroman
