# Supplementary figures and images for: UPLC-MS based urine untargeted metabolomic analyses to differentiate bladder cancer from renal cell carcinoma
Source: BMC Cancer. 2019 Dec 5;19:1195. doi: 10.1186/s12885-019-6354-1 (PMC6896793; doi:10.1186/s12885-019-6354-1)

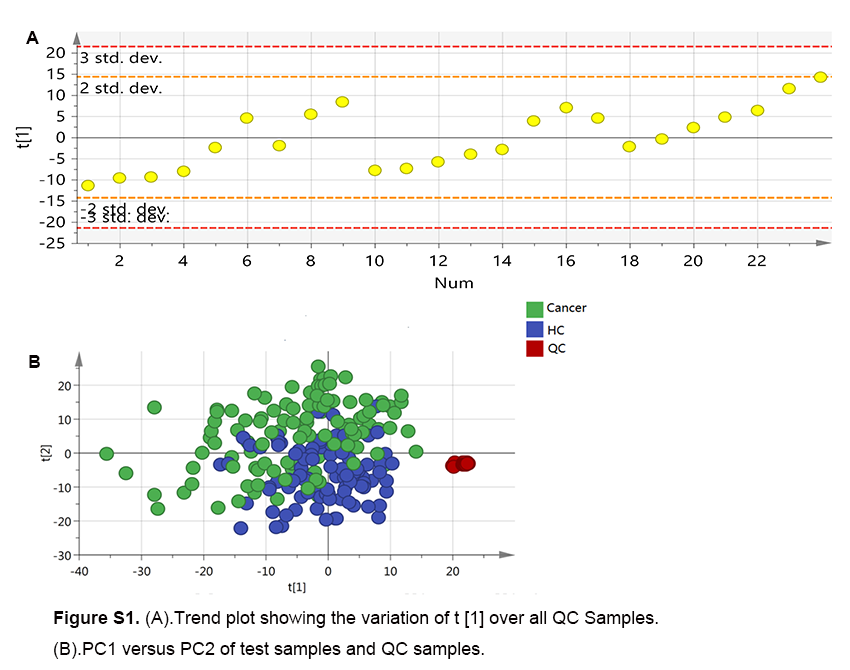

Supplement: Supplementary file 1 — Additional file 1: Figure S1. (A).Trend plot showing the variation of t [1] over all QC Samples.(B).PC1 Versus PC2 of test samples and QC samples. [file 12885_2019_6354_MOESM1_ESM.tif]

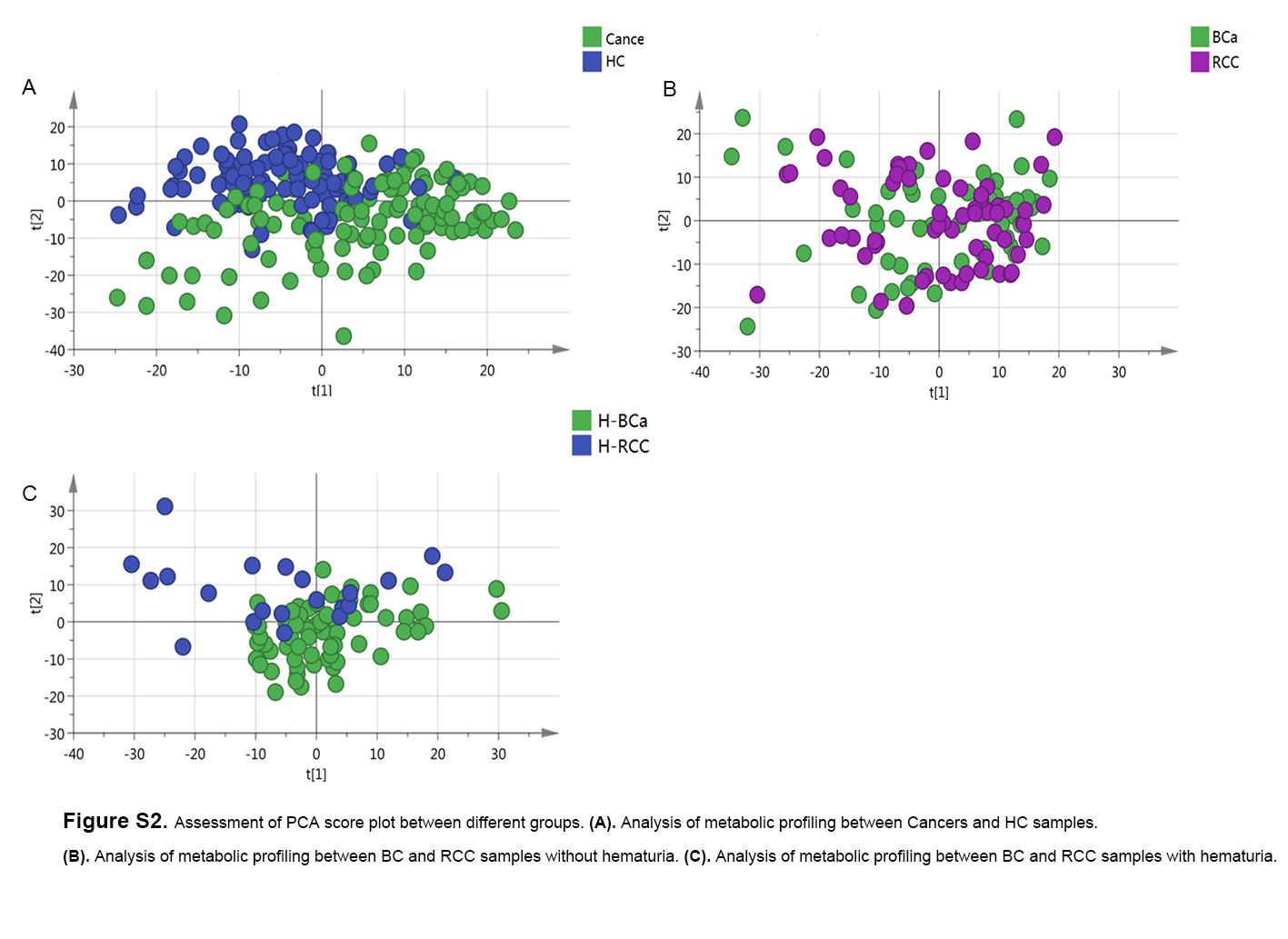

Supplement: Supplementary file 2 — Additional file 2: Figure S2. Assessmentof PCA score plot between different groups. (A). Analysis of metabolic profiling between Cancers and Healthy controls. (B). Analysis of metabolic profiling between BC and RCC samples without hematuria. (C). Analysis of metabolic profiling between BC and RCC samples with hematuria. [file 12885_2019_6354_MOESM2_ESM.tif]
