# Supplementary material for: UPLC-MS based urine untargeted metabolomic analyses to differentiate bladder cancer from renal cell carcinoma
Source: BMC Cancer. 2019 Dec 5;19:1195. doi: 10.1186/s12885-019-6354-1 (PMC6896793; doi:10.1186/s12885-019-6354-1)
Supplement: Supplementary file 3 — Additional file 3: Table S1a. Differential metabolites between cancer(BC and RC) and healthy controls. Table S1b. Differential metabolites for cancer(BC and RC) distinction. Table S2a. Differential metabolites between BC and RC without hematuria. Table S2b. Differential metabolites for BC and RC without hematuria distinction. Table S3a. Differential metabolites between BC and RC with hematuria. Table S3b. Differential metabolites for BC and RC with hematuria distinction [file 12885_2019_6354_MOESM3_ESM.docx]

**Supplement Table 1a. Differential metabolites between cancer(BC and RC) and health control**

| **Variables** | **Metabolites ID** | **Description** | **Score** | ***p*-value** | **Fold Change（cancer/HC）** |
| --- | --- | --- | --- | --- | --- |
| 2.41_269.1593m/z | HMDB40152 | 3-Mercapto-1-hexanol | 38.4 | 2.42E-04 | 1.90 |
| 3.62_265.0838m/z | HMDB02381 | N-Acetylcystathionine | 44.4 | 4.67E-03 | 1.50 |
| 4.39_167.0554m/z | HMDB01886 | 3-Methylxanthine | 37.8 | 1.78E-02 | 0.59 |
| 4.94_253.0916m/z | HMDB00071 | Deoxyinosine | 41.5 | 1.54E-13 | 0.34 |
| 5.01_356.1321m/z | HMDB41191 | Hydrojuglone glucoside | 37.7 | 9.17E-05 | 6.49 |
| 5.73_328.1008m/z | HMDB10316 | Acetaminophen glucuronide | 39.1 | 7.57E-05 | 0.38 |
| 5.85_143.0727n | HMDB40042 | 3-[(5-Methyl-2-furanyl)methyl]-1H-pyrrole | 41.2 | 3.91E-07 | 1.52 |
| 5.85_314.1944m/z | HMDB38120 | Ovalicin | 40.4 | 8.78E-08 | 8.27 |
| 5.85_363.2238n | HMDB61062 | 7-hydroxygranisetron | 41.6 | 5.81E-08 | 1.76 |
| 6.01_258.1089n | HMDB32527 | 1,1'-(Tetrahydro-6a-hydroxy-2,3a,5-trimethylfuro[2,3-d]-1,3-dioxole-2,5-diyl)bis-ethanone | 40.6 | 5.80E-07 | 1.96 |
| 6.05_330.2254m/z | HMDB15026 | Granisetron | 40.4 | 8.72E-03 | 0.29 |
| 6.08_262.0940n | HMDB15058 | Levosimendan | 41 | 1.97E-11 | 1.70 |
| 6.23_283.1135m/z | HMDB32857 | Glycerol tripropanoate | 40.7 | 1.45E-09 | 2.62 |
| 6.31_292.1560m/z | HMDB39660 | Gossyvertin | 42 | 1.58E-03 | 0.58 |
| 6.38_260.1358n | HMDB01518 | Alpha-CEHC | 38.7 | 1.85E-12 | 4.38 |
| 6.45_107.0848m/z | HMDB31414 | 2,3-Dimethyl-2-cyclohexen-1-one | 40.6 | 1.15E-09 | 1.72 |
| 7.22_277.2145m/z | HMDB36091 | exo-5,6-Dimethylbicyclo[2.2.1]hept-5-en-2-ol | 39 | 1.19E-09 | 4.82 |
| 7.36_399.1987m/z | HMDB36340 | Ethyl 7-epi-12-hydroxyjasmonate glucoside | 35.5 | 4.09E-09 | 4.65 |
| 7.42_452.2460m/z | HMDB14326 | Flunisolide | 43.4 | 5.09E-10 | 4.11 |
| 7.44_364.2226n | HMDB06760 | 11b,17a,21-Trihydroxypreg-nenolone | 43.3 | 4.37E-12 | 0.57 |
| 7.44_540.2535n | HMDB10357 | Tetrahydroaldosterone-3-glucuronide | 47.5 | 2.90E-11 | 0.58 |
| 7.69_349.2351m/z | HMDB13221 | Beta-Cortolone | 38.3 | 2.18E-12 | 0.44 |
| 7.72_360.2722m/z | HMDB40901 | 13-Hydroxy-9-methoxy-10-oxo-11-octadecenoic acid | 45.6 | 3.52E-06 | 0.66 |
| 7.72_454.2619m/z | HMDB14984 | Flurandrenolide | 41.2 | 2.39E-08 | 3.79 |
| 7.93_296.1840m/z | HMDB14333 | Esmolol | 43.4 | 9.85E-09 | 5.14 |
| 7.98_342.1711m/z | HMDB32671 | (E)-2',4,4'-Trihydroxy-3-prenylchalcone | 47.3 | 3.28E-11 | 0.52 |
| 8.06_256.1481n | HMDB38051 | alpha-Terpinyl anthranilate | 43.9 | 1.65E-15 | 0.55 |
| 8.13_462.2434n | HMDB29349 | Neryl rhamnosyl-glucoside | 42.2 | 6.69E-08 | 2.53 |
| 8.30_277.2146m/z | HMDB35877 | (Z)-13-Hexadecenoic acid | 47 | 1.08E-06 | 3.70 |
| 8.30_488.2591n | HMDB38732 | alpha-Ionol O-[arabinosyl-(1->6)-glucoside] | 40.9 | 5.01E-07 | 3.22 |
| 8.40_268.2619m/z | HMDB31336 | (Z)-9-Cycloheptadecen-1-one | 38.3 | 9.22E-03 | 0.40 |
| 8.40_269.1731m/z | HMDB38154 | 3,11,12-Trihydroxy-1(10)-spirovetiven-2-one | 44 | 2.51E-06 | 0.36 |
| 8.44_344.1870m/z | HMDB36409 | 1,2-Dihydrodehydroguaiaretic acid | 41.1 | 4.85E-14 | 0.52 |
| 8.51_330.2620m/z | HMDB13321 | Undecanoylcarnitine | 49.7 | 5.10E-05 | 0.38 |
| 8.71_368.2774m/z | HMDB11541 | MG(0:0/18:4(6Z,9Z,12Z,15Z)/0:0) | 40.5 | 5.16E-07 | 0.32 |
| 8.72_344.2775m/z | HMDB31006 | 1-Acetoxy-2-hydroxy-16-heptadecen-4-one | 44.6 | 1.52E-02 | 0.32 |
| 9.00_384.2126n | HMDB36863 | Anhydrocinnzeylanol | 44.5 | 8.60E-08 | 1.58 |

**Supplement Table 1b. Differential metabolites for cancer(BC and RC) distinction**

| **Metabolites** | **AUC** |
| --- | --- |
| Alpha-CEHC | 0.86351 |
| Deoxyinosine | 0.82915 |
| 1,2-Dihydrodehydroguaiaretic acid | 0.81188 |
| Beta-Cortolone | 0.809 |
| E-2',4,4'-Trihydroxy-3-prenylchalcone | 0.80569 |
| alpha-Terpinyl anthranilate | 0.8049 |
| Flunisolide | 0.80063 |
| 11b,17a,21-Trihydroxypreg-nenolone | 0.80054 |
| Glycerol tripropanoate | 0.79862 |
| 3,11,12-Trihydroxy-110-spirovetiven-2-one | 0.79845 |
| exo-5,6-Dimethylbicyclo2.2.1hept-5-en-2-ol | 0.79775 |
| Flurandrenolide | 0.79566 |
| Tetrahydroaldosterone-3-glucuronide | 0.79226 |
| Levosimendan | 0.78519 |
| Esmolol | 0.77534 |
| Ethyl 7-epi-12-hydroxyjasmonate glucoside | 0.77411 |
| MG00/1846Z,9Z,12Z,15Z/00 | 0.77098 |
| 2,3-Dimethyl-2-cyclohexen-1-one | 0.74952 |
| Neryl rhamnosyl-glucoside | 0.74429 |
| 7-hydroxygranisetron | 0.7435 |
| 1,1'-Tetrahydro-6a-hydroxy-2,3a,5-trimethylfuro2,3-d-1,3-dioxole-2,5-diylbis-ethanone | 0.73208 |
| Ovalicin | 0.72955 |
| Anhydrocinnzeylanol | 0.72449 |
| Z-9-Cycloheptadecen-1-one | 0.72065 |
| Z-13-Hexadecenoic acid | 0.71934 |
| alpha-Ionol O-arabinosyl-1-6-glucoside | 0.71703 |
| Granisetron | 0.71655 |
| Undecanoylcarnitine | 0.71193 |
| 3-5-Methyl-2-furanylmethyl-1H-pyrrole | 0.71071 |
| 1-Acetoxy-2-hydroxy-16-heptadecen-4-one | 0.71019 |

**Supplement Table 2a. Differential metabolites between BC and RC without hematuria**

| **Variables** | **Metabolites ID** | **Description** | **Score** | ***p*-value** | **Fold Change（BC/RC）** |
| --- | --- | --- | --- | --- | --- |
| 2.19_248.1477m/z | HMDB13127 | Hydroxybutyrylcarnitine | 47.7 | 8.21E-03 | 1.56 |
| 2.94_112.0862m/z | HMDB14417 | Betazole | 39.7 | 1.29E-02 | 7.92 |
| 4.49_100.1115m/z | HMDB31404 | Cyclohexylamine | 37.9 | 1.02E-04 | 0.07 |
| 4.51_129.0361m/z | HMDB40237 | Dihydro-2-methoxy-2-methyl-3(2H)-thiophenone | 37.1 | 1.99E-02 | 0.53 |
| 4.71_146.0593m/z | HMDB01424 | 4-(3-Pyridyl)-3-butenoic acid | 40.5 | 1.48E-02 | 0.60 |
| 4.85_329.1689m/z | HMDB33939 | Domoic acid | 44.5 | 4.66E-03 | 2.24 |
| 5.35_254.1134m/z | HMDB01200 | N'-Formylkynurenine | 38.6 | 1.77E-02 | 1.93 |
| 5.95_274.1454m/z | HMDB30178 | (S)-Edulinine | 40.1 | 2.69E-02 | 0.18 |
| 5.99_400.2153m/z | HMDB33237 | 1,2,10-Trihydroxydihydro-trans-linalyl oxide 7-O-beta-D-glucopyranoside | 43.5 | 1.78E-02 | 6.67 |
| 6.05_376.1349n | HMDB33300 | (1RS,2RS)-Guaiacylglycerol 1-glucoside | 45.2 | 4.39E-02 | 2.28 |
| 6.30_164.0696m/z | HMDB12883 | Adrenochrome o-semiquinone | 37.1 | 2.42E-02 | 0.58 |
| 6.41_274.0828n | HMDB30486 | (S)-2,3-Dihydro-6-hydroxy-5-(hydroxyacetyl)-2-isopropenylbenzofuran | 39.6 | 1.39E-02 | 1.57 |
| 6.54_272.1841m/z | HMDB61633 | 3-hydroxyhexanoyl carnitine | 41.2 | 7.42E-03 | 1.51 |
| 6.54_388.2307m/z | HMDB32842 | 5-Megastigmen-7-yne-3,9-diol 3-glucoside | 42.8 | 3.41E-02 | 2.14 |
| 6.57_461.1052m/z | HMDB41745 | Hesperetin 7-O-glucuronide | 43 | 4.05E-02 | 2.35 |
| 6.82_263.0504m/z | HMDB01131 | Iminoaspartic acid | 38.9 | 4.60E-02 | 0.64 |
| 6.85_273.0742m/z | HMDB29253 | 3-Hydroxyphloretin | 42.2 | 3.40E-04 | 9.56 |
| 6.89_258.1564n | HMDB32681 | 10alpha-4,5-Seco-11-eudesmene-4,5-dione | 45 | 1.06E-02 | 1.79 |
| 6.89_449.1051m/z | HMDB41728 | (-)-Epicatechin 3'-O-glucuronide | 39.7 | 1.88E-03 | 9.75 |
| 6.92_268.1165m/z | HMDB41857 | Citrinin | 38.4 | 2.43E-02 | 0.14 |
| 6.95_107.0848m/z | HMDB59905 | Ethylbenzene | 42.3 | 1.39E-04 | 1.76 |
| 6.95_135.0797m/z | HMDB39956 | 4-(Ethoxymethyl)phenol | 43.2 | 2.77E-06 | 3.21 |
| 7.04_192.0644m/z | HMDB60400 | 5-Phenyl-1,3-oxazinane-2,4-dione | 39.3 | 2.46E-02 | 0.35 |
| 7.15_305.1838m/z | HMDB40699 | Feruperine | 38.7 | 4.64E-02 | 0.43 |
| 7.25_225.1471m/z | HMDB38736 | (3S,5R,6R,7E)-3,5,6-Trihydroxy-7-megastigmen-9-one | 46.1 | 1.03E-03 | 0.49 |
| 7.26_430.2941m/z | HMDB30057 | Boviquinone 4 | 45.1 | 3.86E-02 | 0.56 |
| 7.28_595.3453m/z | HMDB04159 | L-Urobilin | 47.7 | 2.33E-02 | 0.39 |
| 7.35_312.2144m/z | HMDB13202 | 6-Keto-decanoylcarnitine | 52 | 1.17E-02 | 1.57 |
| 7.35_386.2513m/z | HMDB05099 | Thromboxane B3 | 41.7 | 3.71E-03 | 2.22 |
| 7.35_444.3078m/z | HMDB06318 | Gamma-linolenyl carnitine | 40.1 | 3.02E-02 | 0.47 |
| 7.84_442.1806n | HMDB34120 | Lusitanicoside | 42.6 | 4.17E-02 | 1.63 |
| 8.20_248.1761n | HMDB33917 | 4-Hydroxy-3-methoxy-2,10-bisaboladien-9-one | 50.8 | 1.12E-02 | 1.81 |
| 8.45_372.2723m/z | HMDB01483 | Prostaglandin F2b | 40.4 | 4.30E-03 | 3.30 |

**Supplement Table 2b. Differential metabolites for BC and RC without hematuria distinction**

| **Metabolites** | **AUC** |
| --- | --- |
| 4-Ethoxymethylphenol | 0.72524 |
| Ethylbenzene | 0.71816 |
| Cyclohexylamine | 0.71492 |
| 3-Hydroxyphloretin | 0.68352 |
| Domoic acid | 0.66907 |
| 3S,5R,6R,7E-3,5,6-Trihydroxy-7-megastigmen-9-one | 0.66421 |
| Prostaglandin F2b | 0.6564 |
| Thromboxane B3 | 0.65124 |
| Hydroxybutyrylcarnitine | 0.65035 |
| N'-Formylkynurenine | 0.64682 |
| 3-hydroxyhexanoyl carnitine | 0.64534 |
| --Epicatechin 3'-O-glucuronide | 0.6449 |
| 4-Hydroxy-3-methoxy-2,10-bisaboladien-9-one | 0.64239 |
| 4-3-Pyridyl-3-butenoic acid | 0.63502 |
| 10alpha-4,5-Seco-11-eudesmene-4,5-dione | 0.63031 |
| 6-Keto-decanoylcarnitine | 0.62706 |
| Dihydro-2-methoxy-2-methyl-32H-thiophenone | 0.62176 |
| 5-Phenyl-1,3-oxazinane-2,4-dione | 0.61999 |
| S-2,3-Dihydro-6-hydroxy-5-hydroxyacetyl-2-isopropenylbenzofuran | 0.61778 |
| Betazole | 0.61616 |
| 1,2,10-Trihydroxydihydro-trans-linalyl oxide 7-O-beta-D-glucopyranoside | 0.61306 |
| Citrinin | 0.61262 |
| L-Urobilin | 0.61114 |
| Feruperine | 0.60908 |
| Lusitanicoside | 0.60407 |
| Iminoaspartic acid | 0.60348 |
| Adrenochrome o-semiquinone | 0.6023 |
| Hesperetin 7-O-glucuronide | 0.60127 |
| Boviquinone 4 | 0.60083 |

**Supplement Table 3a. Differential metabolites between BC and RC with hematuria**

| **Variables** | **Metabolites ID** | **Description** | **Score** | **Fold Change（BC/RC）** | ***p*-value** |
| --- | --- | --- | --- | --- | --- |
| 1.81_136.0520n | HMDB32603 | 2-Hydroxy-4-methylbenzaldehyde | 40.9 | 0.43 | 2.02E-05 |
| 1.91_158.1535m/z | HMDB35916 | (E)-2-Nonen-4-one | 39.1 | 2.95 | 4.84E-05 |
| 2.15_387.1391m/z | HMDB60494 | N-Acetylmuramoyl-Ala | 42.1 | 0.36 | 3.75E-05 |
| 2.29_239.1013n | HMDB13642 | 1-hydroxy-2-Oxopropyl tetrahydropterin | 52.2 | 0.50 | 3.76E-06 |
| 2.41_370.1714m/z | HMDB04448 | Estradiol-17beta 3-sulfate | 42.6 | 2.33 | 8.14E-03 |
| 2.56_164.0561m/z | HMDB00802 | Pterin | 42.1 | 0.42 | 9.20E-04 |
| 2.81_152.0562m/z | HMDB00403 | 2-Hydroxyadenine | 50.4 | 0.45 | 1.38E-04 |
| 3.36_300.1071m/z | HMDB14585 | Entecavir | 39.9 | 2.05 | 1.76E-02 |
| 3.50_268.1035m/z | HMDB61067 | arabinofuranosylguanine | 44.2 | 0.45 | 3.78E-04 |
| 4.24_160.0753m/z | HMDB12490 | 1,2-Dehydrosalsolinol | 43 | 0.47 | 1.57E-04 |
| 4.31_239.0996m/z | HMDB29069 | Threoninyl-Proline | 38.7 | 4.73 | 4.09E-02 |
| 4.62_107.0487m/z | HMDB59712 | 3-Hydroxybenzyl alcohol | 43.8 | 0.49 | 1.36E-04 |
| 4.70_191.5898n | HMDB12214 | Dihydrozeatin-O-glucoside | 40.9 | 3.89 | 1.26E-03 |
| 4.72_302.0631n | HMDB06211 | 2-(Formamido)-N1-(5-phospho-D-ribosyl)acetamidine | 41.4 | 3.57 | 2.31E-02 |
| 4.73_256.1173m/z | HMDB11721 | Trans-2, 3, 4-Trimethoxycinnamate | 41.6 | 2.47 | 2.44E-05 |
| 4.79_440.2129m/z | HMDB15433 | Cilazapril | 41.9 | 0.50 | 2.34E-03 |
| 4.93_170.0444m/z | HMDB00439 | 2-Furoylglycine | 41.4 | 2.44 | 3.28E-04 |
| 5.04_181.0716m/z | HMDB02825 | Theobromine | 43 | 5.35 | 3.05E-05 |
| 5.05_285.1261m/z | HMDB28913 | Isoleucyl-Methionine | 41.3 | 0.34 | 6.64E-04 |
| 5.08_334.1852m/z | HMDB35136 | Glucosyl (E)-2,6-Dimethyl-2,5-heptadienoate | 42 | 0.36 | 3.44E-04 |
| 5.14_272.1234m/z | HMDB39845 | 3-Oxooctanoic acid glycerides | 39.2 | 2.03 | 2.67E-03 |
| 5.25_295.1646m/z | HMDB29108 | Tyrosyl-Isoleucine | 39.8 | 0.35 | 6.76E-05 |
| 5.33_155.0699m/z | HMDB00341 | 2-Octenedioic acid | 42.5 | 0.35 | 6.35E-03 |
| 5.33_292.1207m/z | HMDB03306 | Phloretin | 40.5 | 0.44 | 4.49E-05 |
| 5.49_332.1809m/z | HMDB15334 | Nadolol | 40.1 | 2.16 | 4.77E-04 |
| 5.68_372.1049n | HMDB41723 | Dihydroferulic acid 4-O-glucuronide | 43.7 | 2.48 | 3.39E-02 |
| 5.81_156.1014m/z | HMDB40174 | 4-Ethyl-1,2-benzenediol | 37.7 | 0.49 | 3.08E-03 |
| 5.84_293.1158m/z | HMDB11741 | Gamma-Glutamyltyrosine | 41.5 | 0.38 | 1.10E-04 |
| 5.84_423.1786m/z | HMDB35689 | Melleolide | 41.5 | 0.41 | 4.30E-04 |
| 5.86_394.1697m/z | HMDB34207 | 6'-Apiosyllotaustralin | 37.7 | 3.17 | 1.43E-02 |
| 5.89_318.1362m/z | HMDB30875 | 2',4-Dihydroxy-4',6'-dimethoxychalcone | 45.3 | 0.44 | 3.83E-05 |
| 6.07_260.1256n | HMDB32857 | Glycerol tripropanoate | 38.6 | 2.16 | 1.91E-03 |
| 6.10_408.1024n | HMDB41759 | Naringenin 4'-O-glucuronide | 50.6 | 2.04 | 4.55E-02 |
| 6.15_164.0702m/z | HMDB00158 | L-Tyrosine | 45.5 | 0.42 | 3.90E-05 |
| 6.27_129.0655m/z | HMDB00641 | L-Glutamine | 44.3 | 0.49 | 4.75E-03 |
| 6.27_281.1126m/z | HMDB01107 | 7-Methylguanosine | 38.7 | 0.50 | 1.74E-02 |
| 6.45_251.0801n | HMDB60901 | Clozapine glucuronide | 46.4 | 7.36 | 2.02E-02 |
| 6.52_274.2005m/z | HMDB36143 | Monomenthyl succinate | 46.7 | 0.41 | 1.48E-05 |
| 6.65_263.0513m/z | HMDB60011 | N-acetyl-S-(N-allylthiocarbamoyl)-L-cysteine | 40.3 | 2.31 | 3.00E-02 |
| 6.67_137.0593m/z | HMDB30570 | Sylvopinol | 41.5 | 0.47 | 4.04E-05 |
| 6.78_135.0801m/z | HMDB39956 | 4-(Ethoxymethyl)phenol | 42.6 | 3.06 | 7.76E-03 |
| 6.84_107.0851m/z | HMDB59905 | Ethylbenzene | 41.6 | 4.43 | 1.84E-04 |
| 6.85_310.1409n | HMDB29777 | Cymorcin monoglucoside | 44.3 | 5.43 | 2.38E-02 |
| 6.85_342.2630m/z | HMDB31007 | 1-Acetoxy-2-hydroxy-16-heptadecyn-4-one | 40.4 | 2.89 | 5.61E-08 |
| 6.93_384.2231m/z | HMDB39975 | (1S,2S,4R,8S)-p-Menthane-1,2,8,9-tetrol 2-glucoside | 44.4 | 0.46 | 5.71E-04 |
| 7.03_280.1417n | HMDB33465 | Feruloyl-2-hydroxyputrescine | 44.9 | 3.18 | 6.08E-03 |
| 7.11_432.1983n | HMDB60897 | Diphenhydramine N-glucuronide | 41.7 | 2.13 | 2.76E-04 |
| 7.18_151.0749m/z | HMDB38925 | (4-Hydroxy-3-methoxyphenyl)ethanol | 40.9 | 0.47 | 3.53E-03 |
| 7.18_312.2156m/z | HMDB13202 | 6-Keto-decanoylcarnitine | 52.6 | 4.93 | 2.68E-02 |
| 7.25_452.2476m/z | HMDB14326 | Flunisolide | 43.7 | 2.08 | 2.07E-03 |
| 7.51_268.1304n | HMDB12267 | N-Succinyl-L,L-2,6-diaminopimelate | 38 | 4.05 | 3.35E-03 |
| 7.56_269.1893m/z | HMDB38957 | Citronellyl cinnamate | 38 | 0.49 | 1.47E-04 |
| 7.76_401.2159m/z | HMDB36715 | Matsutakic acid A | 41.6 | 2.21 | 5.65E-04 |
| 8.04_466.1961m/z | HMDB03324 | Biotripyrrin-b | 47 | 0.44 | 3.78E-02 |
| 8.13_506.2945m/z | HMDB38732 | alpha-Ionol O-[arabinosyl-(1->6)-glucoside] | 49.6 | 2.39 | 2.23E-02 |
| 8.16_364.2473m/z | HMDB29561 | (+)-Calycanthine | 39 | 2.20 | 6.69E-03 |
| 8.38_345.0863m/z | HMDB33457 | Flazine methyl ether | 47.1 | 0.42 | 3.08E-02 |
| 8.51_285.2570m/z | HMDB30482 | Cardanolmonoene | 38.9 | 0.23 | 6.93E-04 |
| 8.94_432.2582m/z | HMDB30895 | (4R,5S,7R,11x)-11,12-Dihydroxy-1(10)-spirovetiven-2-one 12-glucoside | 37.6 | 2.58 | 3.77E-03 |

**Supplement Table 3b. Differential metabolites for BC and RC with hematuria distinction**

| **Name** | **AUC** |
| --- | --- |
| arabinofuranosylguanine | 0.84817 |
| 1-hydroxy-2-Oxopropyl tetrahydropterin | 0.84058 |
| 2-Hydroxy-4-methylbenzaldehyde | 0.81781 |
| 1-Acetoxy-2-hydroxy-16-heptadecyn-4-one | 0.79986 |
| Monomenthyl succinate | 0.7971 |
| Ethylbenzene | 0.79641 |
| 1,2-Dehydrosalsolinol | 0.78951 |
| Methionyl-Isoleucine | 0.77985 |
| 2',4-Dihydroxy-4',6'-dimethoxychalcone | 0.77778 |
| L-Tyrosine | 0.77709 |
| N-Acetylmuramoyl-Ala | 0.77295 |
| Trans-2, 3, 4-Trimethoxycinnamate | 0.77157 |
| Citronellyl cinnamate | 0.76743 |
| Sylvopinol | 0.76743 |
| Theobromine | 0.76225 |
| 2-Hydroxyadenine | 0.7619 |
| 3-Hydroxybenzyl alcohol | 0.75914 |
| 2-Furoylglycine | 0.75707 |
| Gamma-Glutamyltyrosine | 0.75431 |
| Glycerol tripropanoate | 0.73982 |
| E-2-Nonen-4-one | 0.73844 |
| Pterin | 0.73706 |
| Nadolol | 0.73361 |
| Cilazapril | 0.73223 |
| N-Succinyl-L,L-2,6-diaminopimelate | 0.73154 |
| Dihydrozeatin-O-glucoside | 0.73016 |
| Cymorcin monoglucoside | 0.72533 |
| Melleolide | 0.72464 |
| 3-Oxooctanoic acid glycerides | 0.72257 |
| 1S,2S,4R,8S-p-Menthane-1,2,8,9-tetrol 2-glucoside | 0.71705 |
| Matsutakic acid A | 0.71636 |
| Glucosyl E-2,6-Dimethyl-2,5-heptadienoate | 0.71222 |
| 4-Hydroxy-3-methoxyphenylethanol | 0.71084 |
| Diphenhydramine N-glucuronide | 0.70876 |
| Cardanolmonoene | 0.70807 |
| L-Glutamine | 0.70117 |
