# Supplementary material for: UPLC-MS based urine untargeted metabolomic analyses to differentiate bladder cancer from renal cell carcinoma
Source: BMC Cancer. 2019 Dec 5;19:1195. doi: 10.1186/s12885-019-6354-1 (PMC6896793; doi:10.1186/s12885-019-6354-1)
Supplement: Supplementary file 4 — Additional file 4. Data processing using Progenesis QI [file 12885_2019_6354_MOESM4_ESM.docx]

**Supplemental Materials**

**Data processing using Progenesis QI**

The detailed workflow for data processing facilitated by Progenesis QI is involved “create a new experiment”, “import data”, “review alignment”, “experiment design setup”, “peak picking”, “reviewed convolution--normalization”, and “identify compounds” in sequence. In general, the whole process ran automatically using optimized parameter settings. (1) In the stage of create a new experiment, adduct ion was carefully selected as it would influence the number of characterized compounds and also the identification accuracy. Based on the ionization behaviors of reference standards, the adduct ion forms, comprising [M + H]+, [M + Na]+, [M + K], [M + NH4]+, [2M + H]+, [2M + Na]+,[2M + NH4]+, [M + H – H2O]+ and [M + H – 2H2O]+ , were selected. (2) The MS data acquired by LC-MS for all the URINE samples were imported into the Progenesis QI software, generating a 2D ion intensity map with the retention time and m/z information as the ordinate and abscissa, respectively. (3) Peak alignment was carried out in automatic manner taking a QC run as the reference, the score values for all the samples were greater than 90 %. (4) For peak picking, the thresholds of chromatographic peak absolute intensity, and retention time limits can be set to achieve the maximum real ion signals with noise excluded. In the present study, absolute intensity and retention time limit were set at 1000 and default. “Normalize to all compound” was used to normalized peaks to eliminate sampling and analysis bias. (5) Further compound identification was performed by searching the HMDB database (2017 version). The identification results combined with the intensity data were exported as .csv files for subsequent compound confirmation and multivariate statistical analysis.

**Confirmation of compounds characterization**

Detailed compound identification information (.csv file) included compound ID, adducts, formula, score, fragmentation score, mass error (in ppm), isotope similarity, theoretical isotope distribution, web link, and m/z values. The data was further analyzed in detail, under which more abundant MS/MS fragments were acquired. Confirmation of the differential compounds was performed by the parameters, including Score, Fragmentation score, and Isotope similarity given by Progenesis QI. Score ranging from 0 to 60, is used to quantify the reliability of each identity. According to the score results of the reference standards, the threshold was set at 35.0. Fragmentation score represents the matching degree between the theoretical fragments and the measured ones. The fragmentation score of 0 indicates no match occurs or the compound generates no fragments. Isotope similarity is calculated by comparison of the measured isotope distribution of a precursor ion with the theoretical. The compound identification is more reliable the higher the values obtained.
